# Imbalances in the German public health system - numbers of state-certified occupational physicians and relation to socioeconomic data

**DOI:** 10.1186/s12995-016-0136-3

**Published:** 2016-10-12

**Authors:** Christoph Gyo, Michael Boll, Dörthe Brüggmann, Doris Klingelhöfer, David Quarcoo, David A. Groneberg

**Affiliations:** The Institute of Occupational Medicine, Social Medicine and Environmental Medicine, School of Medicine, Goethe University Frankfurt, Theodor-Stern-Kai 7, 60590 Frankfurt, Germany

**Keywords:** Public health administration, Occupational health

## Abstract

**Background:**

State-certified occupational physicians who work as civil servants in the Federal Republic of Germany are key players in the German Public Health system. They control i.e. the legal compliance in occupational health and participate in the occupational disease procedures. Despite the role model function of the German Public health system for many developing countries, this area of Public health is debated to have been hampered in the past years by a disregard concerning structural developments.

**Methods:**

Different databases were screened for occupational health benchmarks. Obtained data were compared to socioeconomic data and indices were calculated.

**Results:**

The overall numbers of State-certified occupational physicians decreased in Germany between 1992 and 2012 from 136 to 86 (63 %). On the single state level, the ratios of State-certified occupational physicians per 1 Mio. working population ranged from 8 for the state of Saarland to 0.8 for the state of North Rhine Westphalia. A general difference was found for old versus new German states. Also, large differences were present for the ratios of State-certified occupational physicians per 10^6^ employees towards public debt per capita (€) and the ratios of State-certified occupational physicians per Gross Domestic Product (GDP) in the 16 German states in 2012.

**Conclusions:**

In striking contrast to the WHO document on the Occupational safety and health (OSH) system that states in its executive summary that the human and institutional capacities of the German occupational health system are very strong in both quantity and quality, we here show extreme imbalances present at the single state levels that developed over the past 20 years. With a regard to the increasing complexity of the economic system a reversal of this trend should be demanded.

## Background

The German social security system consists of the five pillars of health, pension, accident, long-term care and unemployment insurance. It covers more than 90 % of the German population. Within this system, the occupational health system operates along the conventions of the International Labour Organization (ILO). As in other countries, the system is under current review [1].

A recent WHO document entitled “Country Profile of Occupational Health System in Germany” elegantly summarizes the settings of the German Occupational Health System. It describes that health and safety at work is administered by the Ministries for Labour and Social Affairs at both federal and the level of the 16 German states, thus reflecting the federal structure of Germany [2]. The federal ministry for Labour and Social Affairs (BMAS) has the responsibility within the federal government for health and safety at the federal German level. It is supported by advisory committees on occupational health including i.e. occupational diseases, hazardous chemical substances, biological agents. On the level of the single states, state labour inspection authorities are responsible for implementing Occupational safety and health (OSH) legislation at the state level. There is also an interplay with the statuary accident insurances in this area of supervision and inspection that leads to the term of the “dual OSH system of Germany”.

A key player of the state supervision and inspection was established in the State-certified occupational physician (Gewerbearzt) with a long lasting history in Germany. The first state-certified occupational physician dates back to 1905 in Württemberg, Alsace and Lorraine (German territories in 1905) [3], 1906 (Baden) and 1909 (Bavaria, Franz Koelsch). The first Prussian state-certified occupational physician was established in 1921 in Düsseldorf (Ludwig Teleky). In 1939, 40 State-certified occupational physicians were present in Germany. The WHO document on the Occupational safety and health (OSH) system states in its executive summary that the human and institutional capacities of the system are very strong in both quantity and quality [2]. Consequently, this should be reflected by the numbers of State-certified occupational physicians since they play an important role within the system.

We here hypothesize that the development and number of State-certified occupational physicians in the 16 states of Germany and overall in Germany is not reflecting this. In order to address this issue, numbers of State-certified occupational physicians were searched and related to different socio-economic and accidents features.

## Methods

### Data on state-certified occupational physicians

Data concerning numbers and development of State-certified occupational physicians were retrieved by the use of an internet search. Terms “Gewerbearzt” or “Landesgewerbearzt” were entered in the Google search engine and more than 12000 and 9740 results respectively were obtained. The top 100 entries were screened. Evolution of numbers of State-certified occupational physicians was collected from the platform of the Federal Institute of Occupational Safety and Health at www.baua.de [4].

### Socioeconomic data

Socioeconomic data were retrieved from the German Federal Statistical Office. This is a federal institution with about 2,600 employees who gather, collect, process, present and analyse statistical information. The head office consisting of seven departments and the office leadership is located in Wiesbaden and the operating platform to retrieve data is www.destatis.de. Data on the numbers of physicians related to inhabitants were retrieved from the platform of the Federal chamber of physicians (Bundesärztekammer) [5].

### Data on occupational accidents

Data concerning numbers and development of fatal occupational accidents were retrieved by the use of an internet search. The term of fatal occupational accidents (“Tödliche Arbeitsunfälle”) was entered to the Google search engine and approx. 228,000 results were found. The top 100 entries were screened and numbers were obtained from two sources [4, 5].

## Results

### Federal data

The overall number of State-certified occupational physicians gives an important insight in the integrity of the system. Analysing these numbers between 1992 and 2012, it is obvious that there is a strong decrease in workforce (Table 1) despite the increasing complexity of occupations. From a starting point in 1992 with 136 State-certified occupational physicians (100 %), the level raises to a maximum of 160 State-certified occupational physicians in 1995 (118 %). Then, the level decreases to lower than 100 % from 2004 onwards. In 2007, the rates decreases to 80 % in comparison to 1992 and in 2012, the rate is 63 %.

**Table 1:** Number of State-certified occupational physicians in Germany between 1992 and 2012 [4]

Year	Number of State-certified occupational physicians	
	Absolute numbers	Numbers in % of 1992
1992	136	100
1993	155	114
1994	157	115
1995	160	118
1996	159	117
1997	158	116
1998	158	116
1999	147	108
2000	148	109
2001	147	108
2002	146	107
2003	147	108
2004	130	96
2005	121	89
2006	110	81
2007	109	80
2008	99	73
2009	95	70
2010	90	66
2011	90	66
2012	86	63

**Fig. 1:**
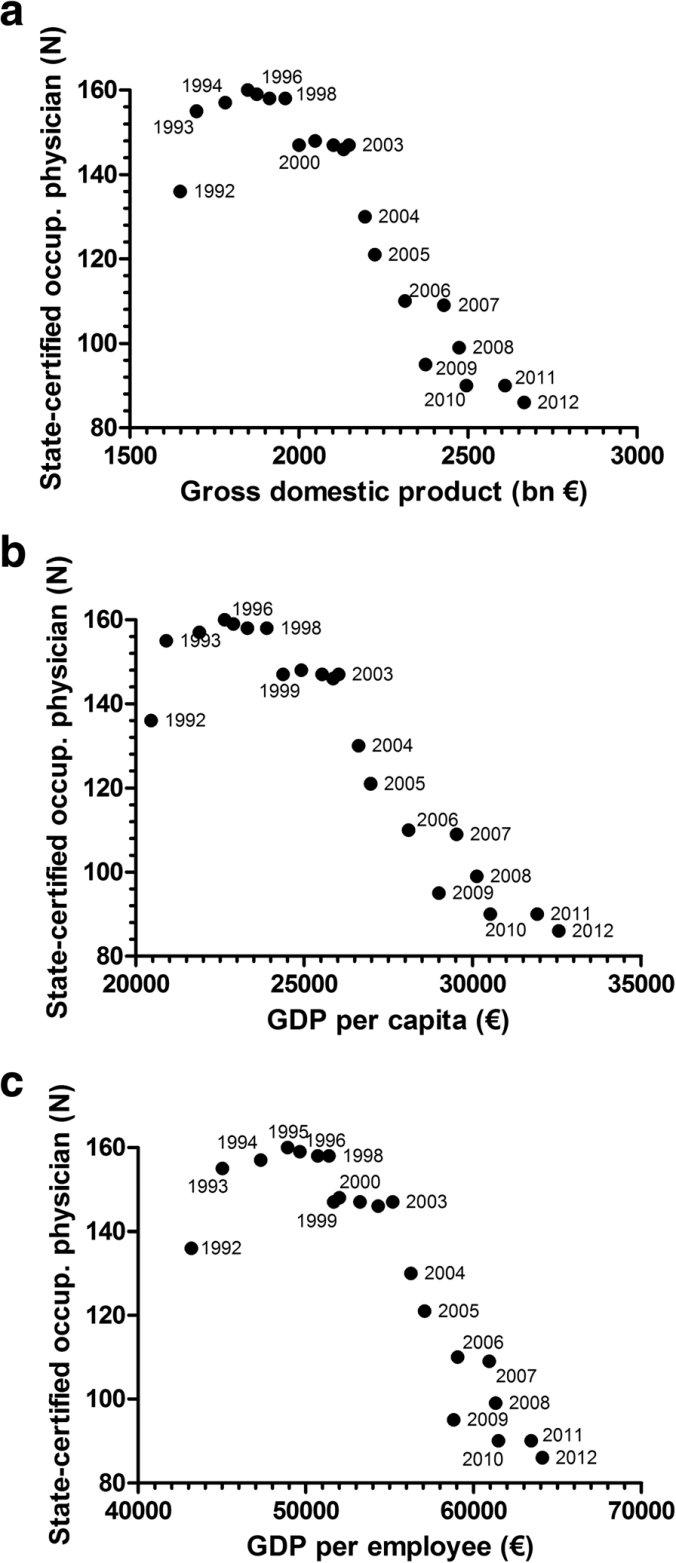
Correlation of State-certified occupational physicians between 1992 and 2012 with the Gross domestic product (GDP in bn €, **a**), the Gross domestic product per capita (**b**), and the Gross domestic product per employee (**c**)

The correlation of the overall numbers of State-certified occupational physicians in Germany between 1992 and 2012 with the Gross domestic product (GDP in bn €) demonstrates that there is a decrease of physician numbers that correlates with an increase in the GDP (Fig. 1a). Similar trends are found when the numbers are correlated with the Gross domestic product per capita, and the Gross domestic product per employee (Fig. 1b and c).

### Single state data

When focussing on the single states, it becomes apparent that there are large differences present between the 16 single German states in 2012. In the total number ranking, Bavaria is ranked 1st with a total of 23 physicians, followed by North Rhine-Westphalia with 7 physicians. The last position is held by Mecklenburg West Pomerania and Bremen, Hamburg, and Schleswig-Holstein with 2 physicians (Table 2).

**Table 2:** State-certified occupational physicians in 2012 in the 16 German states.

#	State	State-certified occupational physicians [23]	Working population (Mio.) (https://www.destatis.de/DE/Startseite.html)	State-certified occupational physicians per 1 Mio. Working population
1	Saarland	4	0.5	8.0
2	Bremen	2	0.4	5.0
3	Brandenburg	5	1.1	4.5
4	Thuringia	4	1.0	4.0
5	Bavaria	23	6.7	3.4
6	Saxony-Anhalt	3	1.0	3.0
7	Berlin	5	1.7	2.9
8	Mecklenburg-West Pomerania	2	0.7	2.9
9	Saxony	5	1.9	2.6
10	Rhineland-Palatinate	4	1.9	2.1
11	Hamburg	2	1.1	1.8
12	Hesse	5	3.1	1.6
13	Schleswig-Holstein	2	1.3	1.5
14	Baden-Württemberg	8	5.6	1.4
15	Lower Saxony	5	3.7	1.4
16	North Rhine Westphalia	7	8.7	0.8

State-certified occupational physicians per 1 Mio. Employees

**Fig. 2:**
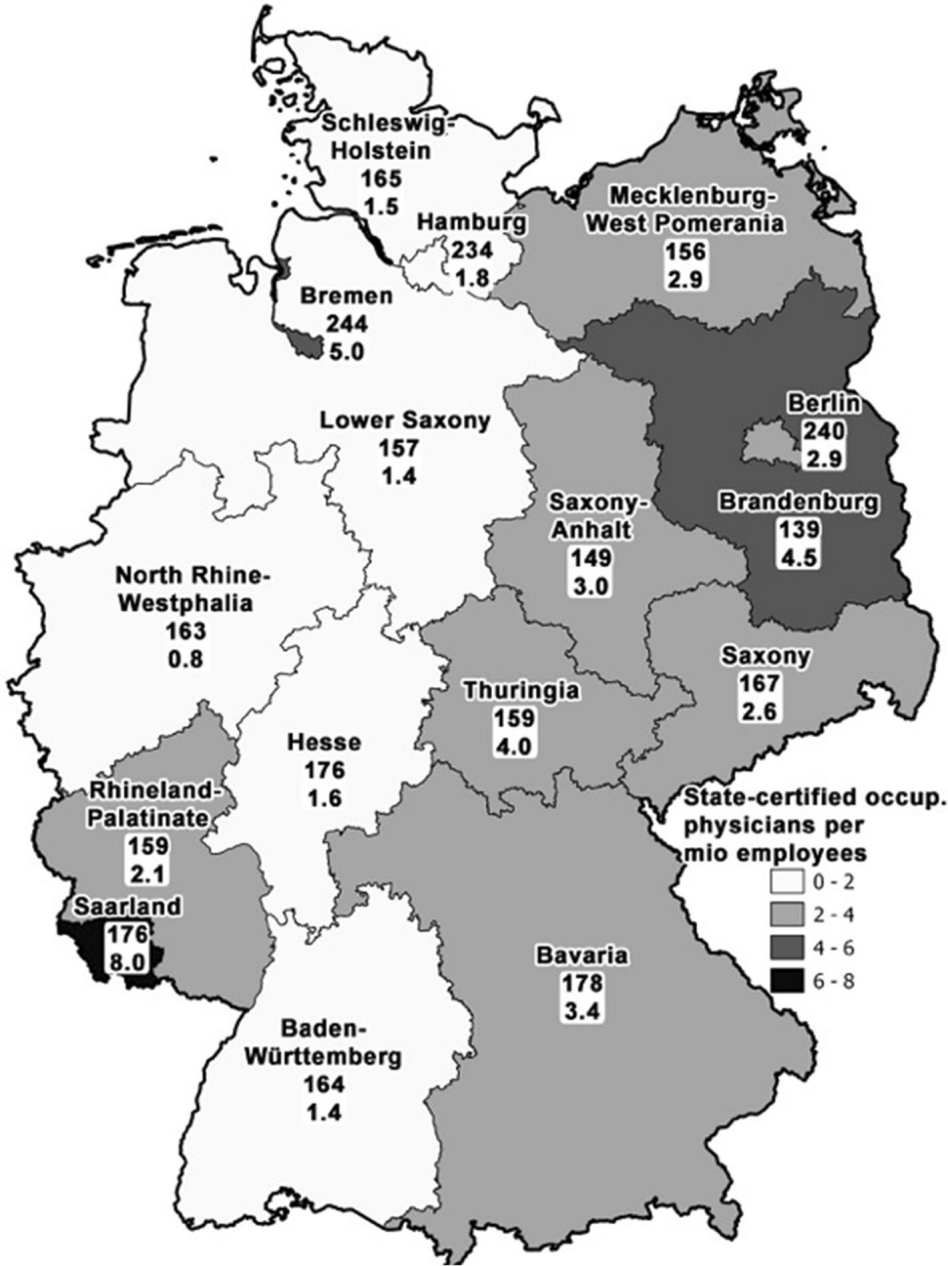
Ratio of State-certified occupational physicians per 1 Mio employees in the 16 German states (*lower ciphers and greyscales*) and ratio of all physicians per 100000 inhabitants per state (*upper ciphers*)

**Fig. 3:**
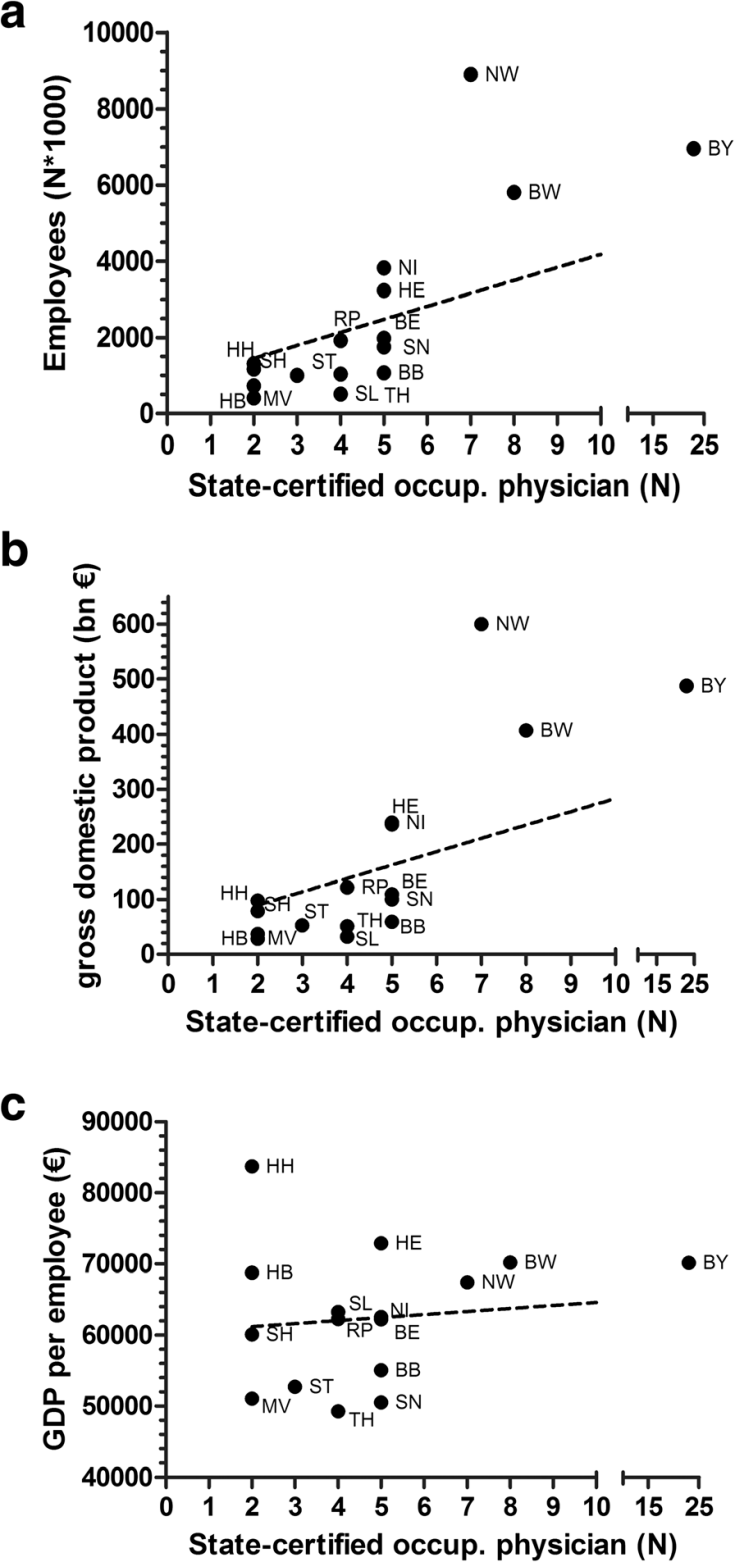
Ratio of State-certified occupational physicians per 1 Mio employees in the 16 German states in 2012 (**a**). Ratio of State-certified occupational physicians per GDP in the 16 German states (**b**). Ratio of State-certified occupational physicians per GDP per employee in the 16 German states (**c**)

The analysis of socioeconomic data in relation to physician numbers changes the ranking. I.e. the ratio of State-certified occupational physicians per 1 Mio employees is calculated at 8 per 1 Mio employees for Saarland, 5 for Bremen, 4.5 for Brandenburg, 4 for Thuringia, 3.4 for Bavaria, 1.4 for Baden-Württemberg and Lower Saxony and only 0.8 for North Rhine-Westphalia (Fig. 2). The two states of North Rhine-Westphalia and Baden Württemberg with relatively large numbers of employees tend to employ less State-certified occupational physicians per employee than the other states (slope +/− SD 340.2 +/− 99.42, *r* ^*2*^ = 0.46, slope significant non-zero (*p* = 0.0041), Fig. 3a). A similar picture is present when the numbers of State-certified occupational physicians are related to the gross domestic product of the single states (slope +/− SD 24.2 +/− 6.9, *r* ^*2*^ = 0.47, slope significant non-zero (*p* = 0.0035), Fig. 3b).

When focussing on the relation of State-certified occupational physicians in single states towards the GDP per employee in single states, no significant relations are found and large variations of the relation are present within the cohort of the 16 states (slope +/− SD 425.6 +/− 491.7, *r* ^*2*^ = 0.05, slope not significant non-zero (*p* = 0.4013), Fig. 3c).

**Fig. 4:**
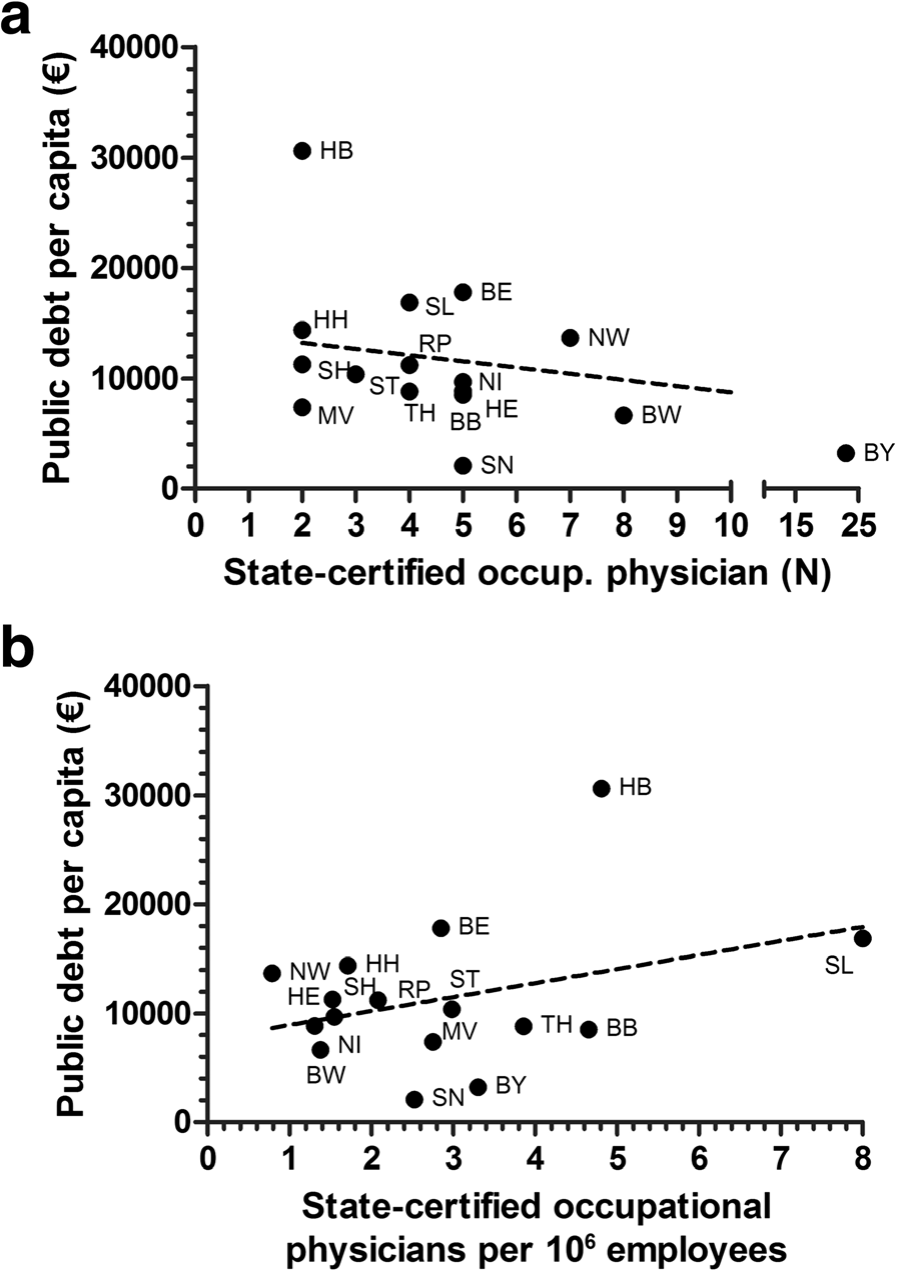
Ratio of public debt per capita (€) in the 16 German states in 2012 towards numbers of State-certified occupational physicians (**a**). Ratio of State-certified occupational physicians per 10 6 employees towards public debt per capita (€) in the 16 German states in 2012 (**b**)

When the public debt per capita in each state is related to numbers of State-certified occupational physicians per state it is found that states with a rather low public debt tend to have relatively high numbers of physicians, i.e. Bavaria or Baden-Württemberg, but there is no significant relation present (slope +/− SD −560.8 +/− 323.4, *r* ^*2*^ = 0.18, slope not significant non-zero (*p* = 0.1049), Fig. 4a).

A comparison of the ratio of State-certified occupational physicians per 10^6^ employees with the public debt per capita shows that the state with the highest number of physicians per employees (SL) has a relatively high public depth per capita. Also Bremen fits into this scheme but there is no overall significant relation present (slope +/− SD −1281 +/− 927.5, *r* ^*2*^ = 0.12, slope not significant non-zero (*p* = 0.1887), Fig. 4b).

## Discussion

This study examines the timely evolution of State-certified occupational physicians in Germany between 1992 and 2012. Furthermore, it analyses the data from 2012 on a single state basis. Overall, it can be summarized that the numbers of State-certified occupational physicians continuously decreases. This tendency is opposed by an increase in the wealth of Germany, as measured by different GDP indices. This increase of wealth is contradictory to the decrease of the numbers of State-certified occupational physicians, since an increase of wealth usually goes along with an increase in tax revenues, which should enable the German States to provide for a stable number of State-certified occupational physicians. Thus, it is unlikely that the decrease of the numbers of State-certified occupational physicians has been necessitated by reasons of the public budget. Such decrease rather seems to be caused by focussing public spending on areas more appealing to voters than workplace safety. This development might have been driven also by a shifting of weights from the industrial sector to the service sector, with “typical” work place injuries like fractures, burns, chemical burns, musculoskeletal diseases etc. being on the decline and less obvious work caused diseases like psychological disorders etc. being on the rise.

**Fig. 5:**
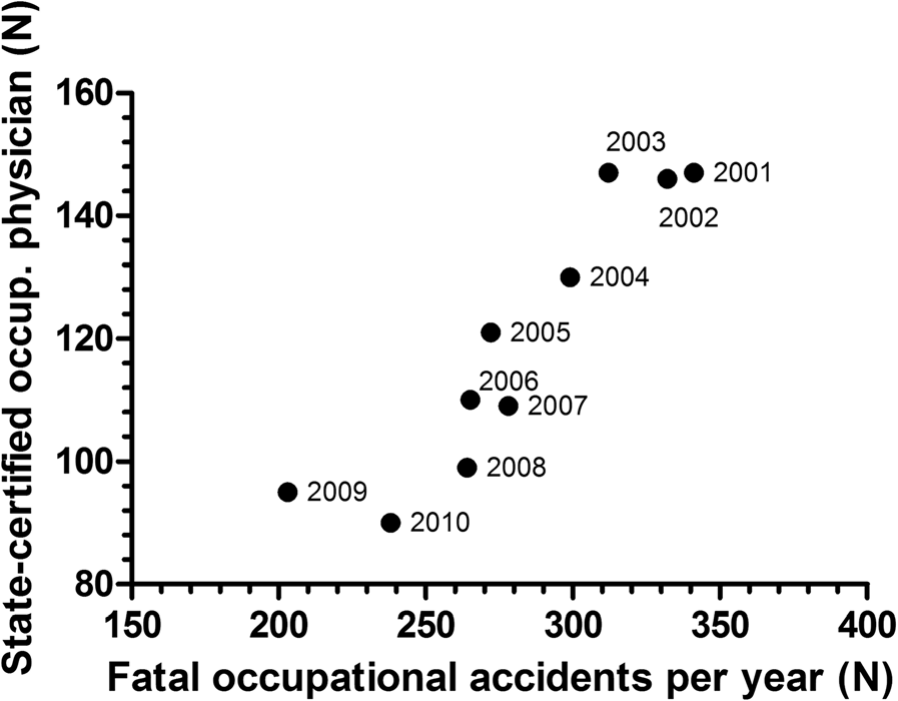
Correlation of State-certified occupational physicians with numbers of fatal occupational accidents

With regard to the decrease in numbers of deadly working accidents (Fig. 5), one might speculate that this decrease may be reasonable due to a lower workload. However, this assumption is wrong since there is a dramatic increase in the complexity of working processes in the industrialized world. I.e. new technologies including the use of nanoparticles [6, 7] or particles in general [8, 9] and infectious diseases [10, 11] in many working surroundings or musculoskeletal issues [12, 13] enforces federal and state authorities to increase the intellectual capacities in this area of the German occupational health system. Also, psychological and lifestyle issues become more and more important in the field [14–21].

When assessing the structure of the deficit in the State-certified occupational physician workforce in Germany one needs to consult single state data. In this respect, the analysis of the ratio of State-certified occupational physicians per 1 Mio employees in the 16 German states and comparison to the ratio of all physicians to inhabitants per state is interesting, since it demonstrates that the decrease of the ratio of State-certified occupational physicians is not reflected by a comparable development in the ratio of all physicians, even though both are part of the public health system and both are indicators for the quality and quantity of healthcare supply for the population. The first index demonstrates a difference between the old Western German states with the exclusion of Bavaria and Rhineland-Palatinate, and the State of Saarland, which was not a founding state of Western Germany, and the new, Eastern German states. It shows that these Eastern German States that belonged to the territory of the communistic German Democratic Republic until 1990 have a higher ratio than the Western states. All Eastern states have numbers of 2 or higher State-certified occupational physicians per million inhabitants whereas the Western countries except Rhineland-Palatinate, Bavaria, and the above mentioned Saarland have numbers of less then 2. This might be due to the longstanding tradition of occupational medicine in the former GDR and a transformation process after 1990 that led to efficient state labour authorities in the new German countries.

Interestingly, the only Western German state that has a unique position is the Saarland. This state however does not belong to the founding states of Western Germany after the Second World War. Prior to its creation as the territory of the Saar Basin by the League of Nations after the First World War, the Saarland did not exist as a unified entity. After the Second World War, it was a French-occupied territory from 1947 to 1956. Between 1950 and 1956, Saarland was a member of the council of Europe. In 1955, the inhabitants were offered independence in another plebiscite. However, they decided that their territory should become a state of the Federal Republic of Germany (Western Germany). Therefore, its current position as the leading German state concerning the ratio of State-certified occupational physician per inhabitant needs to be interpreted on the basis of this history and the wealth of its coal deposits and their large-scale industrial exploitation in the past century. However, the decline of this wealth from the 1980s onwards did not lead to a reduction in the quantity of the occupational health system as measured by the current State-certified occupational physician workforce.

The WHO document on the Occupational safety and health (OSH) system states in its executive summary that the human and institutional capacities of the system are very strong in both quantity and quality [2]. From the present study that revealed extreme imbalances are present in the single state structures of State-certified occupational physicians ranging from about 8 physicians per 1 million employees (Saarland) to 0.8 (North Rhine Westphalia). In an industrialized country such as Germany such inequalities should not be present despite the federal character of the country. Similar trends are also observed for other physicians who serve in the public health area including forensic medicine. Here, since over ten years, a public debate has been started about the shortage of physicians in the area of legal medicine and leading to a reduction of the attractivity to specialize in this area [22].

These inequalities observed here for the Public Health sector are not present in other areas of the German health system. I.e. the juxtaposition of the ratio of State-certified occupational physicians per 1 Mio employees in the 16 German states and the ratio of all physicians to inhabitants per state shows that in the later index which can be regarded as a general index of health system quality, a complete different setting is found (Fig. 2). Here, the federal city-states of Berlin, Hamburg and Bremen have the highest density of physicians with up to 244 physicians per 100,000 inhabitants (Bremen). The territorial states have lower rates but there is not such a gap present as in the ratio of State-certified occupational physicians per 1 Mio. employees in the 16 German states.

With a regard to the increasing complexity of the German economics it should be unanimously demanded that the overall and single state German State-certified occupational physician workforce should be structured along the Saarland as benchmark with a ratio of about 8 State-certified occupational physicians per 1 million employees. The example of the Saarland demonstrates, that it is both possible and desirable to maintain a high ratio of State-certified occupational physicians per employees even though the industrial landscape and the public budget. This would lead to a structure depicted in Table 3 with an overall number of 325 State-certified occupational physicians who could efficiently counsel German companies, control the legal compliance in occupational health, supervision of around 3.006 occupational medicine physicians dealing with employees directly day per day [5] and participate in the occupational disease procedures.

**Table 3:** Proposed numbers of State-certified occupational physicians in the 16 German states with Saarland as benchmark [23]

#	State	Current number of State-certified occupational physicians [23]	Working population (Mio.) (https://www.destatis.de/DE/Startseite.html)	Proposed State-certified occupational physicians
1	Saarland	4	0.5	4
2	Bremen	3	0.4	3
3	Brandenburg	5	1.1	9
4	Thuringia	4	1.0	8
5	Bavaria	23	6.7	54
6	Saxony-Anhalt	3	1.0	8
7	Berlin	5	1.7	14
8	Mecklenburg-West Pommerania	2	0.7	6
9	Saxony	5	1.9	15
10	Rhineland-Palatinate	4	1.9	15
11	Hamburg	2	1.1	9
12	Hesse	5	3.1	25
13	Schleswig-Holstein	2	1.3	10
14	Baden-Württemberg	8	5.6	45
15	Lower Saxony	5	3.7	30
16	North Rhine Westphalia	7	8.7	70

## Conclusions

In summary, the present study identifies inequalities in the occupational health system of the 16 German states of the Federal Republic of Germany by analysing the structure and timely evolution of State-certified occupational physicians. Due to the increase in complexity of the economic system with a multitude of new hazards and technologies, the trend of decreasing numbers of State-certified occupational physicians needs to be stopped and reversed in order to prevent serious structural deficiencies in the German health system.

## Abbreviations

GDP: Gross domestic product
OSH: Occupational safety and health
ILO: International Labour Organization
BMAS: Federal ministry for Labour and Social Affairs
approx.: Approximately
